# Use of serum total protein as a proxy for passive transfer status in lambs

**DOI:** 10.1002/vetr.5435

**Published:** 2025-06-05

**Authors:** Rob F. Kelly, Amy Jennings, Elizabeth Burrough, Geraldine Russell, Katie Adam, Emily Gascoigne, Peers L. Davies, Jennifer Duncan, Andy Hopker, Robert M. Hyde, Fiona M. Lovatt, Ann Bruce, Alexander Corbishley

**Affiliations:** ^1^ Royal (Dick) School of Veterinary Studies and the Roslin Institute University of Edinburgh Midlothian UK; ^2^ APHA Field Services Perth UK; ^3^ Synergy Farm Health Dorchester UK; ^4^ Department of Livestock & One Health Institute of Infection Veterinary and Ecological Sciences University of Liverpool Liverpool UK; ^5^ School of Veterinary Medicine and Science University of Nottingham Sutton Bonnington UK; ^6^ School of Social and Political Science University of Edinburgh Edinburgh UK

## Abstract

**Background:**

Serum total protein (TP) is commonly used to investigate failure of passive transfer of immunity (FPTI) in cattle. This short communication explores the use of serum TP as a proxy of passive transfer (PT) status in lambs.

**Methods:**

Blood samples were obtained from 434 lambs within 8‒24 hours of birth to measure serum immunoglobulin G (IgG) and TP. The relationship between serum IgG and TP was described using a linear regression model. Receiver operating characteristic analysis was used to select a serum TP cut‐off for sub‐optimal PT.

**Results:**

There was a moderate positive relationship between serum IgG and TP (*r* ^2^ = 0.57). The serum TP cut‐off with the optimal sensitivity (86.8%, 95% confidence interval [CI]: 80.8‒92.1%) and specificity (80.2%, 95% CI: 75.3‒84.8%) to detect serum IgG concentrations of 24 mg/mL or less was 6.8 g/dL.

**Limitations:**

This work involved lowland lambs sampled from a single well‐managed farm. Further work is needed to establish FPTI cut‐offs in a variety of sheep populations.

**Conclusions:**

Serum TP measurement is an appropriate field diagnostic to estimate the PT status of lambs in lowland flocks.

## INTRODUCTION

In UK sheep flocks, infectious diseases, such as enterotoxaemia (watery mouth), omphalitis (navel‐ill) and polyarthritis (joint‐ill), can account for 6‒30% of neonatal mortality.^1^, ^2^ Lambs that survive may have a reduced growth rate and require increased feed costs to rear to slaughter.^3^ To protect against these neonatal diseases, it is essential that lambs receive sufficient colostrum within 24 hours of birth.^4^ Failure of passive transfer of immunity (FPTI) occurs when lambs receive insufficient quality or quantity of colostrum in the first 6 hours of life, which results in inadequate absorption of colostral antibodies from the gastrointestinal tract.^5^ The incidence of FPTI is poorly estimated in sheep farming systems globally, partly due to the lack of a consensus definition of FPTI in lambs and limited access to affordable validated diagnostic tests.

In UK dairy and beef cattle systems, evaluation of serum immunoglobulin G (IgG) and total protein (TP) concentrations has been used to estimate FPTI prevalence,^6^, ^7^ and there is good evidence that serum IgG concentration is a predictor of calf outcomes.^8^, ^9^ For example, in beef systems, serum IgG concentrations of 24 g/L or less have been associated with increased rates of treatment or death.^10^ Although serum IgG measurement is the gold standard test to assess passive transfer (PT) status in all relevant species, testing requires laboratory analysis that can be cost prohibitive.^10^ Serum TP is often used as a proxy for serum IgG when investigating PT status in calves^11^, ^12^ because it can be measured cheaply using a refractometer in the field.^13^ Therefore, measurement of serum TP concentration is a commonly used practical tool for monitoring colostrum management and PT status in proactive cattle health planning.^14^

To assess PT status using serum IgG, laboratory fees alone are ∼£11.00 per lamb (2019‒2023 prices), which is cost prohibitive for most sheep farmers. Being able to quickly and cheaply estimate PT status in lambs would allow estimation of the incidence of FPTI. Therefore, understanding the relationship between lamb serum IgG and TP would provide a cost‐effective diagnostic tool to investigate FPTI on individual farms.

In this study, we explore the association between serum IgG and TP in sheep, using samples from a larger longitudinal lamb cohort study.^15^ We present the sensitivity (SE) and specificity (SP) of different TP values for detecting serum IgG concentrations of 24 g/L or less; the serum IgG threshold used to identify sub‐optimal PT in cattle^9^ and associated with an increased risk of reduced daily liveweight gain (DLWG) from birth to weaning in lambs.^15^

## MATERIALS AND METHODS

Lamb serum samples were collected in 2019 as part of a longitudinal study investigating the relationship between maternal metabolic status and lamb outcomes in a lowland flock in Midlothian, UK.^15^ Blood samples were obtained from lambs within 8‒24 hours of birth to measure serum IgG and TP. In addition, a small subset of lambs were blood sampled at birth to estimate serum IgG and TP baseline measurements prior to colostrum ingestion (negative cohort). After centrifugation to remove the blood cells, serum TP was assessed immediately using a digital refractometer as per manufacturer guidelines (MISCO Digital‐Dairy refractometer) and the remainder of the serum was stored at ‒20°C. Once all serum samples were collected, serum IgG was assessed by the Biomarkers Laboratory at Scotland's Rural College using an ovine IgG sandwich ELISA.^16^ Further details of the study design, sampling method and laboratory procedures are described elsewhere.^15^

All data merging, cleaning, analysis and plotting were performed in R Studio 0.98^17^ using the *tidyverse* package.^18^ Numeric variables were summarised by calculating the median and interquartile range (IQR). A scatter plot and a linear regression model were used to describe the relationship between serum IgG and TP. Selection of a positive cut‐off value for serum TP, that optimises test SE and SP, was conducted using receiver operating characteristic (ROC) analysis.^19^, ^20^ The pROC package roc and plot functions were used to conduct the ROC analysis.^21^ Calculation of TP SE and SP should be considered in light of the serum IgG cut‐off value used to define ‘true’ disease status. Although FPTI using serum IgG is poorly defined in lambs, serum IgG concentrations of 24 mg/mL or less are predictive of poorer outcomes in beef calves,^22^ with values above this cut‐off representing adequate PT. In the lambs sampled in this study, we identified that serum IgG concentrations of 24 mg/mL or less were associated with an increased risk of reduced DLWG from birth to weaning.^15^ Consequently, we used 24 mg/mL or less as a cut‐off to estimate the diagnostic performance of serum TP to identify FPTI. Selection of the positive cut‐off value will depend upon the purpose of the diagnostic test; whether to maximise SE or SP or maintain both.^23^ When investigating PT status, a clinician is arguably most interested in a result with a high positive predictive value (PPV). Considering this preference for few false positives, we report two TP cut‐offs: one that maximises SP and another that balances SE and SP.

## RESULTS

**FIGURE 1:**
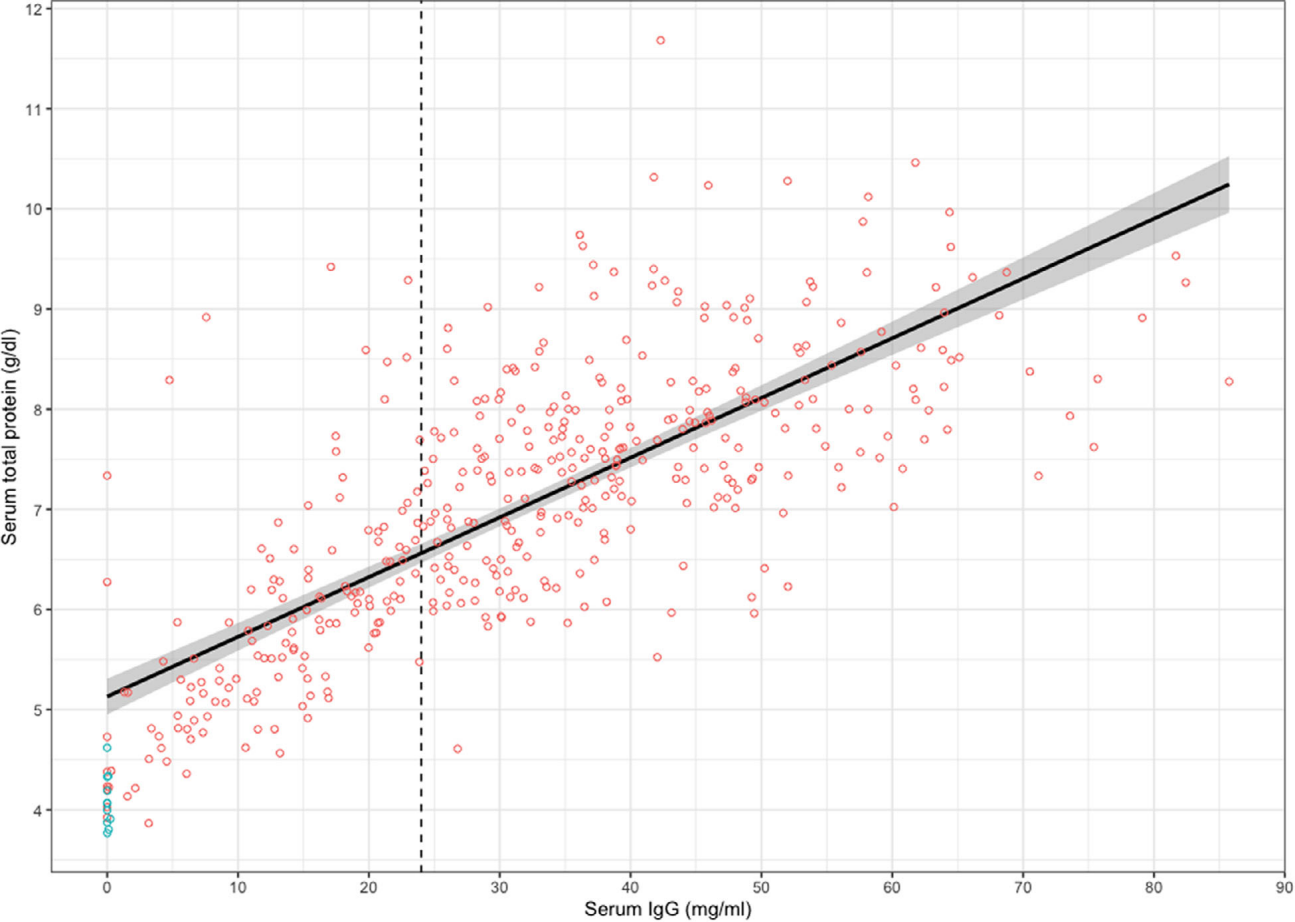
Relationship between serum immunoglobulin G (IgG) and serum total protein in 423 lamb serum samples collected 8‒24 after birth (red) and 11 lamb serum samples collected at birth (blue). Dotted line: serum IgG cut‐off used to identify failure of passive transfer of immunity.

**FIGURE 2:**
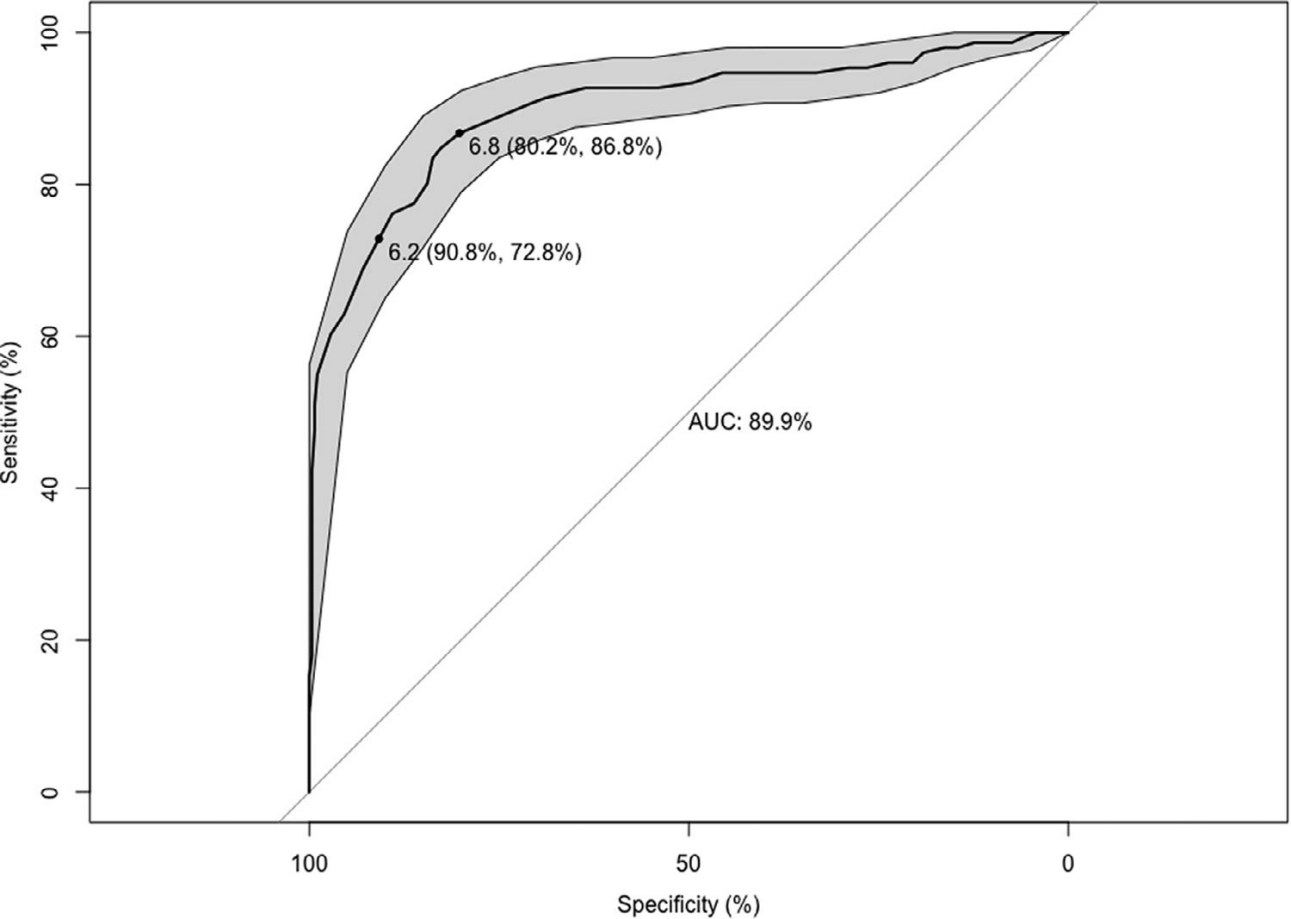
Receiver operating characteristic (ROC) curve for serum total protein (TP) (*n* = 434). The sensitivity (SE) and specificity (SP) of serum TP to detect lambs with a serum immunoglobulin G (IgG) concentration of 24 mg/mL or less are represented on the *y* and *x* axes, respectively. The line represents SE and SP, with the 95% confidence interval (CI) represented by the grey shaded area. The top left‐hand corner of the curve represents the maximum SE and SP. The area under the curve (AUC) represents an aggregate measure of performance across all possible cut‐off values. A TP cut‐off value of 6.8 g/dL balances SE (86.8%) and SP (80.2%), while a TP cut‐off value of 6.2 g/dL prioritises SP (90.8%) over SE (72.9%).

In total, 434 serum samples were collected from lambs at birth (*n* = 11) or 8‒24 hours postpartum (*n* = 423). The serum IgG concentration of samples taken at birth (median = 0.00 mg/mL, IQR = 0.00‒0.05 mg/mL) was lower than for samples taken 8‒24 hours postpartum (median = 31.66 mg/mL, IQR = 19.71‒44.53 mg/mL). A similar relationship was noted for serum TP (at birth: median = 4.10 g/dL, IQR = 3.90‒4.25 g/dL; 8‒24 hours postpartum: median = 7.20 g/dL, IQR = 6.1‒8.00 g/dL). As there was a moderate positive relationship between serum IgG and TP (Figure 1, *r* ^2^ = 0.57), we explored the diagnostic performance of serum TP (in g/dL) using serum IgG concentrations of 24 mg/mL or less as the cut‐off for FPTI. ROC analysis indicated that serum TP was ‘excellent’ (area under the curve: 89.9%) at discriminating between lambs with adequate PT and FPTI when compared to the gold standard serum IgG measurement (Figure 2). The serum TP cut‐off with optimal SE (86.8%, 95% CI: 80.8‒92.1%) and SP (80.2%, 95% CI: 75.3‒84.8%) was calculated as 6.8 g/dL or less. When prioritising SP (90.8%, 95% CI: 87.3‒94.0%) over SE (72.9%, 95% CI: 66.2‒79.9%), the cut‐off for serum TP was 6.2 g/dL or less. As expected, there was a decrease in false positives (26/283 post‐colostral samples >24 mg/mL) using the 6.2 g/dL cut‐off compared with using the 6.8 g/dL cut‐off (56/283). In the study population, this equates to a PPV of 79.2% and 68.2%, respectively. Conversely, there was a decrease in false negatives (20/140 post‐colostral samples ≤24 mg/mL) using the 6.8 g/dL cut‐off compared with using the 6.2 g/dL cut‐off (41/140), equating to a negative predictive value of 91.9% and 86.2%, respectively.

## DISCUSSION

FPTI is potentially a major driver for neonatal disease in UK lowland sheep flocks, yet its incidence is poorly quantified due to a lack of affordable diagnostics.^24^ FPTI may contribute to the practice of lambs being given prophylactic oral antibiotics at birth,^25^ due to increased susceptibility to neonatal infections.^1^ As is the case with cattle, we have demonstrated that serum TP could be a useful proxy for assessing PT status in lambs using simple laboratory equipment. In our study population, a TP cut‐off of 6.2 g/L gave a PPV of 79.2%, that is, ∼80% of lambs with a TP below 6.2 g/L will have a serum IgG concentration of 24 mg/mL or less. We contend that this is sufficient to justify using serum TP for clinical investigation of PT status, for example, sampling batches of lambs during lambing could help identify whether FPTI is contributing to disease and death, hence assisting veterinarians and farmers in deciding whether to take preventive action in future lambing seasons.^26^

In contrast to calves, which are typically blood sampled to determine PT status at 2‒5 days of age, we chose to sample lambs 8‒24 hours after birth. In most indoor lambing flocks, lambs are only penned with their mothers for 24 hours after birth and then released, either into group pens or outside. Therefore, it is impractical to propose blood sampling lambs at 2‒5 days of age due to most being turned out to extensive pasture by this time. Sampling at 8‒24 hours after birth may underestimate final blood IgG concentrations, due to the potential for colostral antibodies to continue to be absorbed after blood sampling; however, the majority of IgG is absorbed quickly after the first feed, which typically occurs well before 8 hours of age. We therefore contend that sampling at this age represents a pragmatic compromise between what may be ideal and what is possible in a commercial environment.

Further work is required to assess whether our reported serum IgG and TP cut‐offs are suitable across different sheep populations, as this study involved lambs from a single, well‐managed farm. Although we have previously identified that twin lambs at risk of reduced DLWG had serum IgG concentrations of 24 mg/mL or less,^15^ we have been unable to investigate the relationship with neonatal mortality and morbidity due to the low prevalence of infectious disease in the study population. A study in dairy lambs in Turkey proposed a lower serum TP threshold of 5.5 g/dL^26^ to define FPTI, and this cut‐off was used in a subsequent UK case report.^27^ This highlights the need for a consensus position as to which serum IgG (and hence TP) cut‐off(s) should be used to define FPTI in sheep, something that has only recently been agreed in cattle.^28^

In conclusion, serum TP measurement shows promise as a field diagnostic to determine the PT status of lambs. Sampling neonatal lambs to assess PT status could be useful to assess the prevalence of FPTI in UK lowland flocks and help set industry benchmarks to support veterinarians in providing evidence‐based individual flock advice to minimise the impact of neonatal disease.

## AUTHOR CONTRIBUTIONS

Alexander Corbishley, Rob F. Kelly, Amy Jennings, Fiona M. Lovatt, Peers L. Davies, Emily Gascoigne, Jennifer Duncan, Robert M. Hyde, Andy Hopker, Katie Adam and Ann Bruce conceived the original project. Alexander Corbishley and Rob F. Kelly designed the field study, sample design and databases associated. Rob F. Kelly, Alexander Corbishley and Amy Jennings developed the field SOPs and collected the data. Rob F. Kelly, Alexander Corbishley and Elizabeth Burrough conducted laboratory work. Rob F. Kelly, Alexander Corbishley and Geraldine Russell cleaned the initial dataset. Rob F. Kelly, Alexander Corbishley and Amy Jennings contributed to the analysis. Rob F. Kelly was responsible for writing the initial drafts. Rob F. Kelly, Amy Jennings, Emily Gascoigne, Peers L. Davies, Jennifer Duncan, Andy Hopker, Robert M. Hyde, Fiona M. Lovatt, Ann Bruce and Alexander Corbishley all contributed to the final draft.

## CONFLICT OF INTEREST STATEMENT

The authors declare no conflicts of interest.

## ETHICS STATEMENT

The work was reviewed and approved by the Animal Welfare & Ethical Review Body at R(D) SVS and conducted under licence in accordance with the Animals Scientific Procedures Act 1986 (ASPA).

## Data Availability

The data that support the findings of this study are available from the corresponding author upon reasonable request.
